# Coalescence of two growing bubbles in a Hele–Shaw cell

**DOI:** 10.1038/s41598-022-05252-5

**Published:** 2022-01-24

**Authors:** Masatoshi Ohashi, Atsushi Toramaru, Atsuko Namiki

**Affiliations:** 1 Department of Earth and Planetary Sciences, Kyushu University, Motooka, Nishi-ku, Fukuoka 819-0395 Japan; 2 Graduate School of Environmental Studies, Nagoya University, Furo-cho, Chikusa-ku, Nagoya 464-8601 Japan

**Keywords:** Fluids, Scaling laws, Fluid dynamics

## Abstract

An understanding of the dynamics of growth-driven coalescence is important in diverse fields across natural science and engineering. Motivated by the bubble coalescence in magma during volcanic eruptions, we study both experimentally and theoretically the coalescence of two growing bubbles in a Hele–Shaw cell. In our system, bubbles grow by gas expansion due to decompression and the diffusional influx of dissolved gas in the liquid. Our experiments show that the evolution of film thickness and bubble shape depends on viscosity, decompression rate, and cell gap. Through a scaling analysis and a perturbation approximation, we find that the hydrodynamic interaction between two bubbles is characterized by a film capillary number $Ca_f=(\eta {\dot{R}}/\sigma )(R/D)^2$ depending on viscosity $\eta$, bubble radius *R*, growth rate ${\dot{R}}$, interfacial tension $\sigma$, and cell gap *D*. The experimental results demonstrate that the film capillary number solely determines the bubble distortion just before coalescence. Under our experimental conditions, bubble coalescence occurs below a critical value of a nominal film capillary number defined as a film capillary number evaluated when two undeformed circular bubbles come into contact.

## Introduction

The coalescence of bubbles is ubiquitous in nature over a wide range of scales. A volcanic eruption is one of the striking examples of the importance of growth-driven bubble coalescence in natural foams. Like a well-shaken cola, volcanic eruption is caused by the sudden exsolution of volatile components dissolved in magma. The explosivity of an eruption depends on the amount of gas in magma that exists as bubbles, and thus the interconnected bubbles formed by coalescence can help to release the gas and prevent the magma from an explosive eruption^1–3^. Bubble coalescence is triggered by several mechanisms, such as the buoyancy force of a bubble and the pressure difference between gas and surrounding liquid^4,5^. In the present study, we focus on the growth-driven coalescence associated with decompression, which is a dominant mechanism of ascending magma. The coalescence of bubbles is driven by an increase in bubble volume due to expansion or a diffusional influx of dissolved gas.

Growth-driven coalescence is not limited to volcanic eruptions but can be seen in polymeric foam formation^6,7^ and dough fermentation^8,9^. In these applications, an understanding of the evolution of bubble size and shape through coalescence is valuable for producing desirable foams because their physical properties are controlled by the bubble structure. For instance, in order to bake a bread with a large volume and a fine crumb texture (e.g., small and thin-cell bubbles with a uniform size), we need dough that prevents bubbles from coalescence during fermentation^10^. Typically, surfactants have a large impact on the stability of a thin film^11^, but for simplicity, we here consider surfactant-free bubbles.

To date, most previous studies on bubble coalescence have focused on the film drainage in a low-viscosity liquid by moving a bubble of constant volume to another bubble or free surface^12–14^. The coalescence in these studies is controlled by inertia force and is different from the growth-driven coalescence in a more viscous liquid, such as magma and polymers. Useful insights into growth-driven coalescence can be obtained through direct observations under controlled conditions. A previous in-situ experiment of growth-driven coalescence used expanding drops^15^. In this experiment, two isolated drops were connected to two opposing capillary tubes and expanded in a more viscous liquid. The design using capillary tubes quantitatively allows us to observe the evolution of film thickness as used in several previous studies^16,17^. However, its main limitation is the connection between the drops and the tubes. A bubble cannot grow and move freely in three-dimensional space. Furthermore, for understanding of foam formation, gas bubbles have to be used because the viscous resistance in a film is sensitive to the boundary condition between the bubble (or drop) and the surrounding liquid.

Another in-situ experimental design used a quasi-two-dimensional cell in which a bubble suspension is clamped with two transparent plates and separated by a small gap^18–22^. In this type of cell, called a Hele–Shaw cell, we can increase the volume of bubbles by physical or chemical foaming and can observe their behaviors directly through the transparent plate. The thin cell makes it possible to observe bubble coalescence without overlapping with another bubble. Since the bubbles can expand and move freely in quasi-two-dimensional space, both non-coalesced and coalesced bubbles can be expected, depending on experimental conditions. Due to the above advantages, the quasi-two-dimensional experiment enables the analysis of coalescence in a more direct and simple manner than the three-dimensional experiment like a drop attached to a capillary tube.

Recently, the coalescence of two drops has been studied experimentally in a Hele–Shaw cell in which liquid drops with a constant volume were made to collide or were separated by an external flow^23^. Theoretical models of drop deformations were proposed based on the perturbation method and lubrication theory^24,25^. These theoretical approaches can be applied to the interfacial deformations of two expanding bubbles.

Here, we investigated, both experimentally and theoretically, the coalescence of two growing bubbles in a Newtonian fluid confined in a Hele-Shaw cell. We find that the deformed bubble shape and the coalescence condition can be understood by a simple scaling and a perturbation solution.

## Experiment

**Figure 1:**
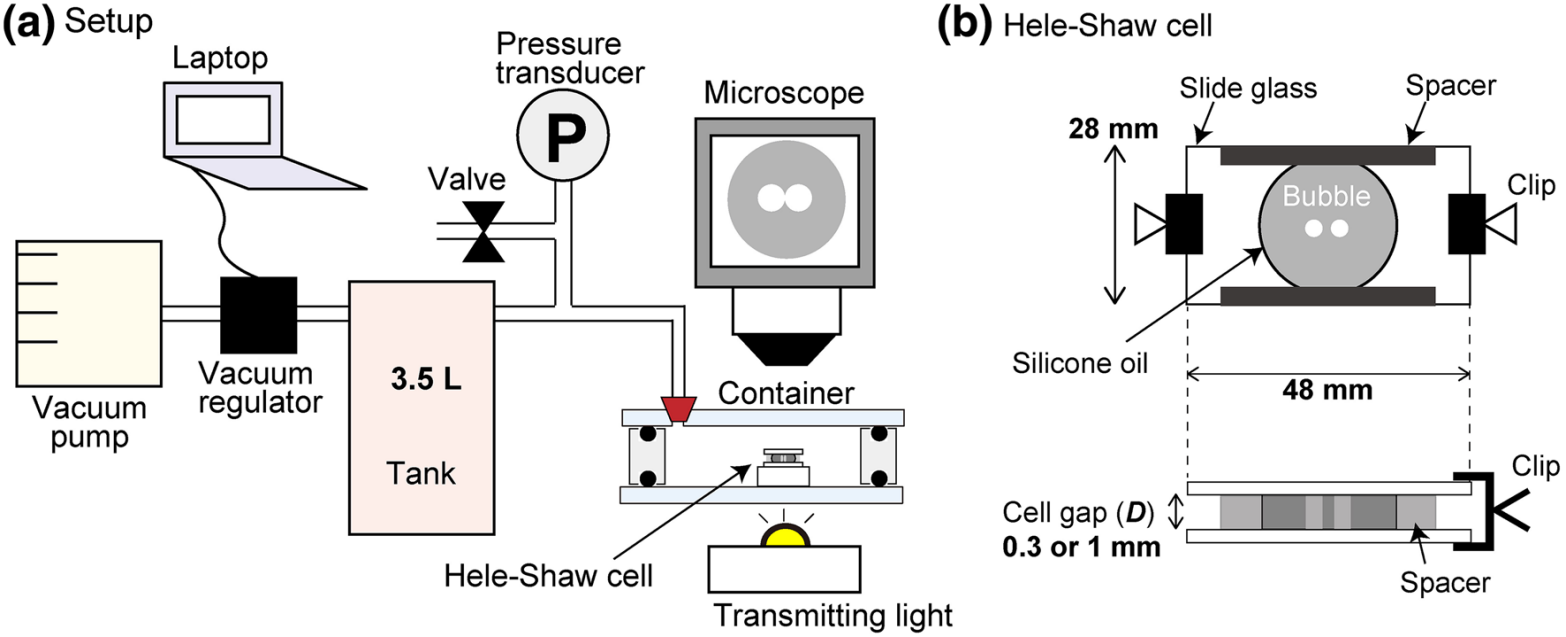
(**a**) Experimental setup. (**b**) Top and side view of the Hele–Shaw cell.

We located the Hele–Shaw cell in a transparent decompression container to minimize the local pressure heterogeneity and controlled the ambient pressure (Fig. 1). The Hele-Shaw cell made from two glass plates is separated by a small distance *D* with uniform spacers. We filled the cell with silicone oil and injected two air bubbles with a microsyringe. When the cell gap was smaller than $1 \ \mathrm{mm}$, injecting two separated bubbles is difficult, so we injected a bubble and then divided the bubble into two smaller bubbles using a needle. We placed the cell in a transparent acrylic container sealed by an O-ring. The interior of the container was decompressed with the vacuum pump from the atmospheric pressure to $10 \ \mathrm{kPa}$. The decompression rate *dp*/*dt* was controlled to be constant by a vacuum regulator until the pressure asymptotically reaches $10 \ \mathrm{kPa}$. After reaching the near-vacuum pressure ($10 \ \mathrm{kPa}$), we kept the pressure constant for $60 \ \mathrm{s}$ and then returned the pressure to atmospheric pressure. The two bubbles gradually approached each other as the bubbles grew and drained out the liquid film between the bubbles. The coalescence process was recorded with an optical microscope and the time evolution of the bubble shape was analyzed using Matlab. We varied the oil viscosity $\eta$ from 1 to $100\ \mathrm{Pa \cdot s}$ and the cell gap *D* from 0.3 to $1.0 \ \mathrm{mm}$. Details of the experimental method are described in the Method section.

## Results

### Growth of a single bubble

**Figure 2:**
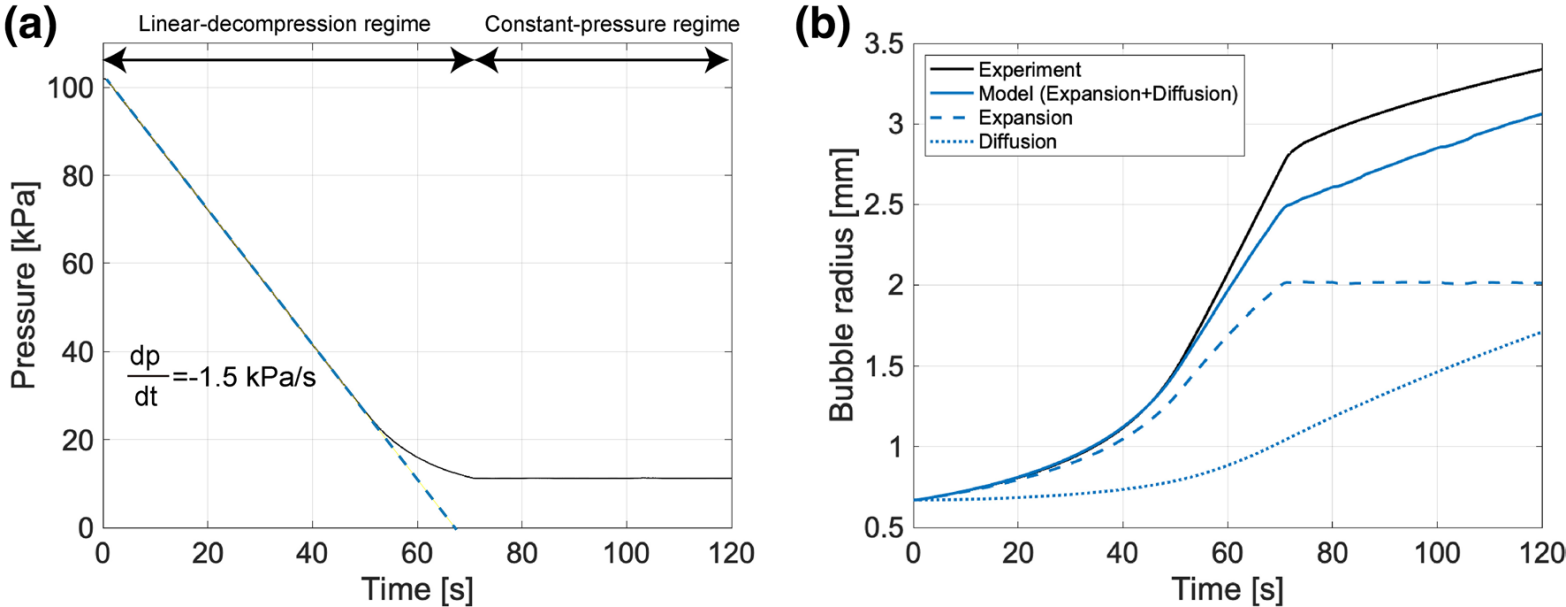
Typical growth of a single bubble during decompression ($dp/dt=-1.5 \ \mathrm{kPa/s}$, $\eta =10 \ \mathrm{Pa \cdot s}$, $D=0.3 \ \mathrm{mm}$). (**a**) Pressure change. The dashed blue line indicates the decompression rate of $dp/dt=-1.5 \ \mathrm{kPa/s}$. (**b**) Bubble radius. The solid black line indicates the experimental result. The solid blue line indicates the model result given by Eq. (S9) in the supplementary information. Dashed and dotted blue lines indicate the model results considering only expansion (first term on the right-hand side of Eq. S9) and only diffusion of dissolved air (second term on the right-hand side of Eq. S9), respectively.

We first investigated the growth rate of a single bubble during decompression. Figure 2 shows examples of the temporal changes of ambient pressure in the container and the effective bubble radius *R*. In the present study, the bubble radius is given by $R=\sqrt{A/\pi }$, where *A* is the projected area of the bubble. In a typical example (Fig. 2a), the pressure decreases at a constant rate of $dp/dt=-1.5 \ \mathrm{kPa/s}$ until $50 \ \mathrm{s}$. The decompression rate slows down slightly as the pressure reaches the final value of $p=10 \ \mathrm{kPa}$. Figure 2b indicates that the bubble radius increases with time and its increase rate, i.e., its growth rate, also increases with time before reaching the final pressure. In our decompression experiments, bubble growth is driven by two mechanisms: gas expansion, which we assume follows behaviour according to the ideal gas law, and the diffusional influx of dissolved air. To understand the contribution of two mechanisms, we made a growth model of a cylindrical bubble placed in a two-dimensional space. The model assumes the ideal gas law and the diffusion equation of dissolved air including $\mathrm{N_2}$, $\mathrm{O_2}$, and $\mathrm{CO_2}$^26^. The interfacial gradient of a dissolved gas in a Hele-Shaw cell is based on a previous study^27^. The assumption of the cylindrical bubble geometry in the Hele-Shaw cell gives the solution of interfacial concentration gradient that has a different coefficient from the solution of the spherical bubble geometry in the three-dimensional space (Eq. S6). See the supplementary information for further information.

When using the above model, we assume that the pressure is uniform through the silicon oil and inside the bubble. The scales of the Laplace pressure and the viscous stress are given by $\sigma / D$ and $\eta R {\dot{R}} /D^2$, respectively, where $\sigma$ is the surface tension and ${\dot{R}}$ is the growth rate. By taking $\sigma$ of around $20 \ \mathrm{mN/m}$, *D* of $0.3 \ \mathrm{mm}$, $\eta$ of $100 \ \mathrm{Pa \cdot s}$, *R* of $1 \ \mathrm{mm}$, and ${\dot{R}}$ of order $0.01 \ \mathrm{mm/s}$, we find the Laplace pressure and the viscous stress around $70 \ \mathrm{Pa}$ and $11 \ \mathrm{Pa}$, respectively. These values are negligible compared to the absolute pressure in the Hele-Shaw cell, and thus the assumption of uniform pressure between liquid and bubble is reasonable in our experiments.

The calculated bubble radius is shown by the solid blue line in Fig. 2b, together with the contribution of expansion (dashed blue line) and diffusional influx (dotted blue line). This demonstrates that the model result has good agreement with the experimental result before reaching the final pressure, and expansion is dominant in the liner decompression region ($t<70 \ \mathrm{s}$). After $t>60 \ \mathrm{s}$, the calculated bubble radius is slightly lower than the experiment. This deviation is much larger than the spatial resolution ($0.012 \ \mathrm{mm/pixel}$) and cannot be explained by error associated with image analysis. We consider that this deviation results from the model deficiencies. The interfacial gradient of the dissolved gas we used does not reflect the advective transfer of dissolved gas due to the interface movement (Eq. S1 in the supplementary information). Moreover, the bubble shape is assumed to be cylindrical, although the air-water interface is curved in our experiments. We should take into account these effects for a more precise model.

### Coalescence of two bubbles

**Figure 3:**
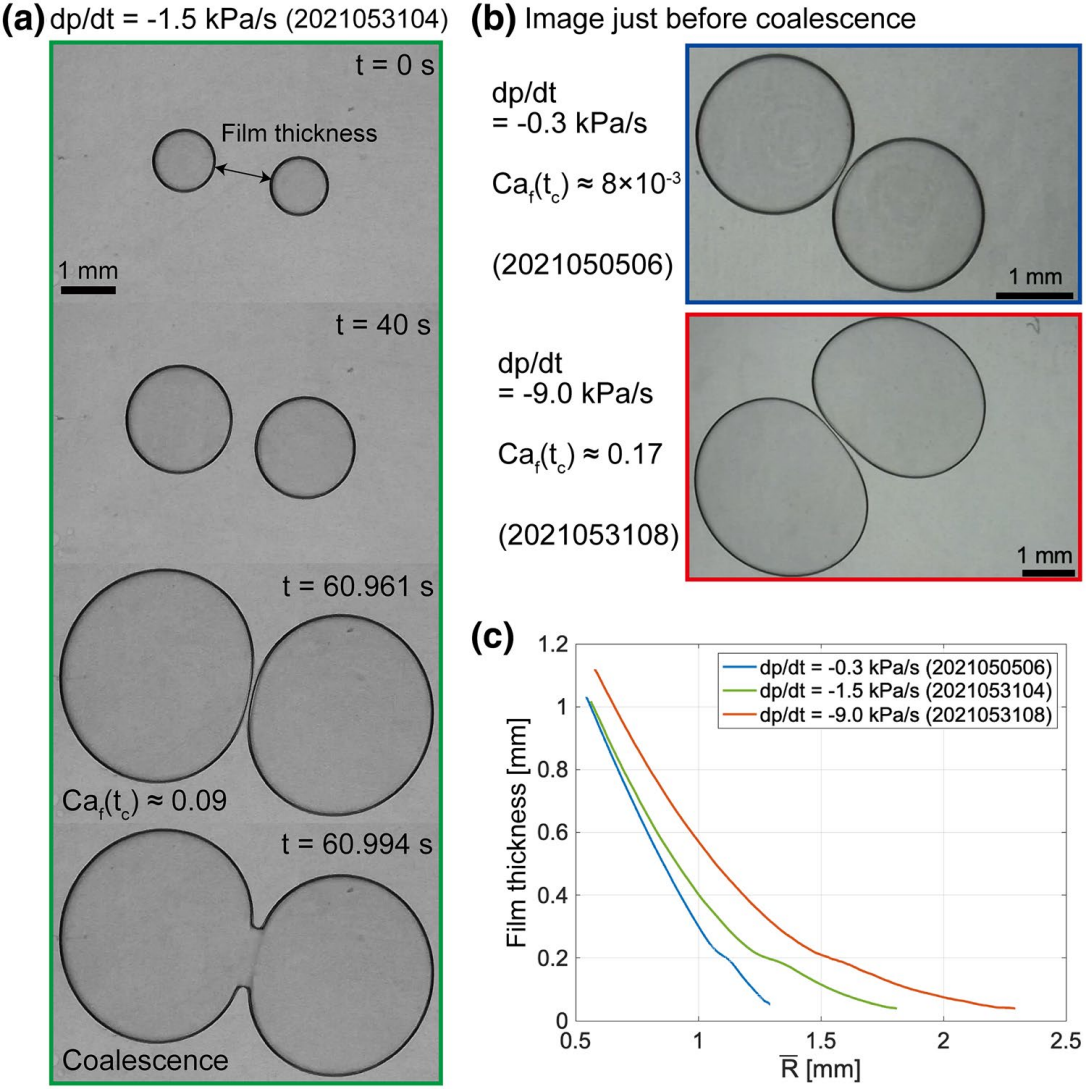
(**a**) Image sequence of bubble coalescence for $D=0.3 \ \mathrm{mm}$, $\eta =1 \ \mathrm{Pa \cdot s}$, and $dp/dt=-1.5 \ \mathrm{kPa/s}$ (2021053104). The film thickness is shown by an arrow. An image taken at $t=60.961 \ \mathrm{s}$ is the bubble shape just before coalescence ($t=t_c$). (**b**) Images just before coalescence. All runs have $\eta =1 \ \mathrm{Pa \cdot s}$ and $D=0.3 \ \mathrm{mm}$, but have different decompression rates. (**c**) The evolution of film thickness with the average radius of two bubbles (${\bar{R}}(t)$). The experiment runs are the same as those shown in (**a**) and (**b**).

We placed two bubbles in a Hele–Shaw cell and observed the evolution of bubble shape. Some examples are shown in Fig. 3a. The vacuum pump reduces the pressure from the atmospheric pressure to $10 \ \mathrm{kPa}$ linearly, as shown in Fig. 2a. When the bubbles become close to each other, they form a thin film between them. The thin film eventually breaks up as the bubbles grow, resulting in coalescence. See also videos in the supplementary materials. Figure 3b shows the bubble shape just before coalescence. Bubbles under slow decompression are almost circular (blue), whereas those under rapid decompression distort and expand parallel to the film (red).

Figure 3c shows the temporal change of a film thickness until bubble coalescence as a function of average bubble radius ${\bar{R}}$. Here, the film thickness is defined as the shortest distance between each interface (Fig. 3a), and the average bubble radius ${\bar{R}}$ is defined as the average of two equivalent bubble radii. In Fig. 3c, the film thickness cannot reach zero, even at the moment just before coalescence. Due to the complex light path, the tips of adjacent bubbles become brighter, and the black area, which is recognized as an interface in the image analysis, moves inward slightly^28^. Therefore, the image analysis overestimates the film thickness when the two bubbles are very close to each other. The three runs in Fig. 3c show the decompression rate dependence under the same viscosity, same gap thickness, approximately the same initial film thickness, and same bubble size. The point of Fig. 3c is that bubbles grow to larger size before coalescing when decompression rate is faster. The fast decompression rate increases the shear stress limiting the film drainage and results in slow film drainage. The bubble expands more as shown in Fig. 3b, so that the bubbles grow to the large size.

**Figure 4:**
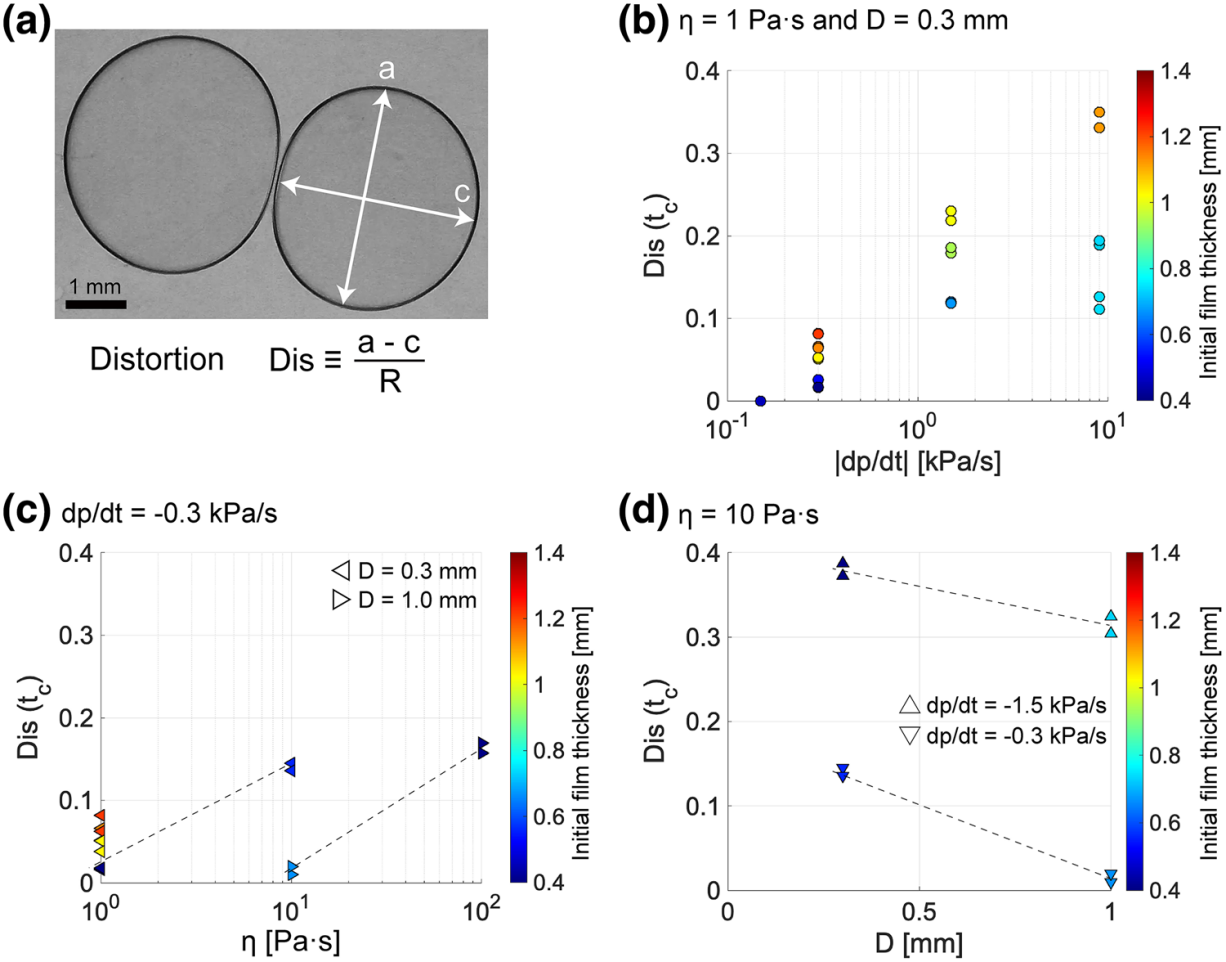
(**a**) Definition of bubble distortion (2021053104). (**b**–**d**) Bubble distortion just before coalescence $Dis(t_c)$ as functions of (**b**) decompression rate $\left| dp/dt \right|$, (**c**) viscosity, and (**d**) cell gap. Marker color indicates the initial film thickness. (**b**) $\eta =1 \ \mathrm{Pa \cdot s}$ and $D=0.3 \ \mathrm{mm}$. (c) $dp/dt=-0.3 \ \mathrm{kPa/s}$. $D=0.3$ or $1.0 \ \mathrm{mm}$. (d) $\eta =10 \ \mathrm{Pa \cdot s}$. $dp/dt=-1.5$ or $-0.3 \ \mathrm{kPa/s}$.

To quantify the distortion, we define the distortion of a bubble *Dis* as

1 $$\begin{aligned} Dis \equiv \frac{a-c}{R}, \end{aligned}$$

where *a* is the maximum Feret diameter, and *c* is the minimum Feret diameter (Fig. 4a). The maximum and minimum Feret diameters correspond to the maximum and minimum lengths, respectively, between two parallel tangents of the bubble. Figure 4b,c,d demonstrate *Dis* as a function of decompression rate, viscosity, and cell gap. Since *Dis* changes with time *t*, we here use *Dis* at the moment just before coalescence $t_c$ as $Dis(t_c)$. We deal with the experiments in which bubbles coalesce only in the linear-decompression region (Fig. 2a) because the pressure history of other bubbles coalesced in the constant-pressure region after the linear decompression is complex. The bubble distortion increases with the decompression rate and viscosity but decreases with the gap of the Hele-Shaw cell. In Fig. 4b, bubbles with thicker initial film thickness are more distorted. For the coalescence of bubbles separated with a greater initial film thickness, the bubbles need to grow more. The bubble size and growth rate increase with time (Fig. 2b), and thus bubbles with larger initial film thickness have larger sizes and growth rates when they get close to each other. The larger size and growth rate lead to the larger viscous force acting on a bubble, as we will discuss in the next chapter. Therefore, the bubbles with larger initial film thickness can be more distorted.

## Theory and its application

The bubble distortion is controlled by the hydrodynamic interactions between two growing bubbles. This problem requires the physical consideration of the competition between viscous forces acting on bubbles and their interfacial tensions, as in the case of bubble deformation in a shear flow^29^. One of the key points is the driving force of the drainage or thinning of the liquid film. In the case of a rising bubble toward the free surface, the film drainage is driven by gravitational and capillary forces^5^. However, the film drainage in our study is triggered by the growth of bubbles. The pressure gradient along the film is determined by the flow flux that is a function of the growth rate. Hereinafter, we describe the bubble distortion in two ways: as a scaling law and as an analytical perturbation solution.

### Scaling analysis

**Figure 5:**
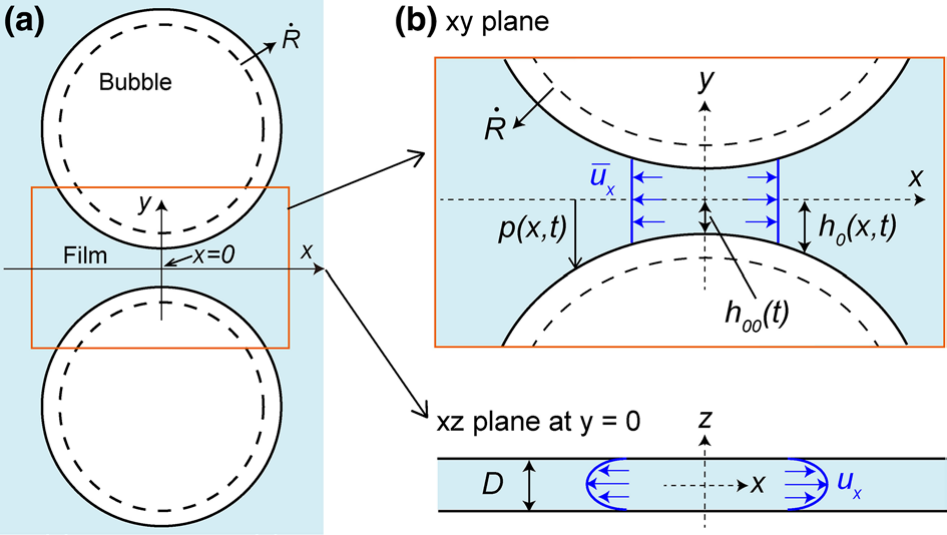
(**a**) Schematic image of two growing bubbles in a Hele-Shaw cell. (**b**) Enlarged views around liquid film in the *xy* and *xz* planes. The bubbles are assumed to be circular, except for $x \ll R$ during growth.

We first seek the order of the viscous force using a lubrication approximation. We consider the film drainage of two growing bubbles in a Hele–Shaw cell. Two bubbles are placed in a Hele–Shaw cell in the *xy* plane with the cell gap *D* in the *z* direction (Fig. 5). The bubbles have the same radius *R* and grow at a growth rate ${\dot{R}}$. In scaling analysis, we assume that the hydrodynamic forces acting on the bubble interface are smaller than surface tension, and thus the bubbles are almost circular. The half-film thickness and its minimum value are expressed by $h_0(x,t)$ and $h_{00}(t)$, respectively (Fig. 5b). When the bubble radius is much larger than the film thickness and the cell gap, i.e., $h \ll R$ and $D \ll R$, respectively, the main contributions to the viscous forces result from a thin lubrication layer around $x=0$. The flow in the film is approximately unidirectional along the *x* axis.

For $x \ll R$, the bubble interface can be approximated by a parabola. The half-film thickness $h_0(x,t)$ is then given by

2 $$\begin{aligned} h_0(x,t) \approx h_{00}(t)+\frac{x^2}{2R}=h_{00}(t) \left( 1 +\frac{x^2}{2R h_{00}(t)} \right) . \end{aligned}$$

We apply the lubrication approximation to our problem, but one might wonder if this application is reasonable because the film thickness obviously reaches the order of $h_0(x,t) \sim R$ at the film end and cannot be regarded as small. As with a previous study^30^, we think that it does not really matter, because the viscous forces mainly come from the very thin film around $x=0$.

One of the problems in the film drainage of growing bubbles is the choice of characteristic length scale. The half-film thickness varies from a minimum value of $h_{00}$ to a maximum value of $h_{00}+R$ along with the *x* axis, and thus it is not entirely clear what choice to make for the characteristic *x*- and *y*-length scales. Here, we assume that the *y*-length scale *Y* has to be $h_{00}$ because the viscous force, which we are interested in, originates from the thin layer. The condition of $Y=h_{00}$ postulates that the curvature term in Eq. (2) satisfies $x^2/2Rh_{00}=O(1)$. Therefore, *x*-length scale *X* has to be

3 $$\begin{aligned} X=\sqrt{Rh_{00}}. \end{aligned}$$

The characteristic velocity can be derived from the continuity equation and the length scales. The continuity equation is given by

4 $$\begin{aligned} \frac{\partial u_x}{\partial x}+\frac{\partial u_y}{\partial y}=0, \end{aligned}$$

where $u_x$ and $u_y$ are the velocities in the *x* and *y* directions, respectively. The *y*-velocity scale $U_y$ corresponds to the bubble growth rate $U_y={\dot{R}}$. We obtain the *x*-velocity scale $U_x$ by equating the order of the first term of Eq. (4) with that of the second term

5 $$\begin{aligned} U_x=\sqrt{\frac{R}{h_{00}}}{\dot{R}}. \end{aligned}$$

Because of $1 \ll R/h_{00}$, Eq. (5) indicates that $U_x$ is much larger than ${\dot{R}}$ ($=U_y$).

The small thickness of a film suggests that the prominent velocity component inside the film is $u_x$. While the interface between the liquid and the glass plate requires the no-slip condition, the interface between gas and liquid phase in the absence of surfactant is expected to be the fully mobile condition^5,13^. From these boundary conditions, the flow in the film becomes a Poiseuille flow across the *xz* plane and like a plug flow in the *xy* plane (Fig. 5b). Through a lubrication approximation assuming the quasi-steady condition, the Stokes equation in the film is given by

6 $$\begin{aligned} \frac{\partial p}{\partial x}=\eta \frac{\partial ^2 u_x}{\partial z^2}, \end{aligned}$$

where *p* is the pressure inside the film. This equation represents the force balance between the pressure gradient from the center to the end of the film and the viscous dissipation inside the liquid film. Given that the *z*-scale length is *D*, the Stokes equation (Eq. 6) and the scales of length and velocity (Eqs. 3 and 5) provide the scale of the viscous pressure in the film $P_{vis}$

7 $$\begin{aligned} P_{vis} = \frac{\eta {\dot{R}}R}{D^2}. \end{aligned}$$

Here, we neglect the pressure in the surrounding liquid. The analytical solution of Eq. (6) also supports Eq. (7) (see Eq. S30 in the supplementary information).

For a circular bubble, the interfacial stress between bubble and liquid (silicone oil) is given by

8 $$\begin{aligned} \tau _{\sigma } = \frac{\sigma }{R}. \end{aligned}$$

Balancing $P_{vis}$ and $\tau _{\sigma }$, we obtain a non-dimensional number referred to as film capillary number $Ca_f$

9 $$\begin{aligned} Ca_f = \frac{P_{vis}}{\tau _{\sigma } }=\frac{\eta {\dot{R}}}{\sigma }\frac{R^2}{D^2}. \end{aligned}$$

The film capillary number defined here is in contrast with the corresponding film capillary number $Ca_f=(\eta V/\sigma ) (R/h_{00})^{3/2}$ for the colliding droplets in a Hele–Shaw cell^25^. Here, *V* is the moving speed of bubbles. The apparent difference of $Ca_f$ between droplets and bubbles is due to the boundary condition of the film. The no-slip condition was applied to the interface between droplet and surrounding liquid in Chan et al.^25^, but the freely mobile condition was applied to the interface between bubble and surrounding liquid in our experiments.

### Perturbation solution

The bubble distortion just before coalescence can also be derived analytically. There have been several theoretical and experimental studies to evaluate the interaction dynamics between drops or bubbles moving to face each other^13,14,31,32^. Among these studies, Lai et al.^24^ and Chan et al.^25^ derived an analytical solution of film thickness between two moving drops confined in a Hele–Shaw cell. Based on the conservation of bubble volume during deformation, the inner solution of bubble shape around the apex was obtained^25^. Here, we extended the method of Chan et al. to the deformations of two growing bubbles confined in a Hele–Shaw cell. We derived the pressure distribution inside a film by a lubrication theory^30^ and then applied this distribution to the augmented Young–Laplace equation^33^. The analytical perturbation solution of the bubble distortion can be obtained in the limit where viscous forces acting on a bubble are much smaller than its interfacial tensions. Details of our model are described in the supplementary information. When $h_{00}(t)$ is much smaller than the bubble radius, the dependence of the bubble distortion on $h_{00}$ can be approximately neglected. By $h_{00} \rightarrow 0$, the analytical solution of the bubble distortion just before coalescence $Dis(t_c)$ is simplified as follows:

10 $$\begin{aligned} Dis(t_c) = \left( \frac{265}{6}-\frac{38 \pi }{3} \right) Ca_f(t_c). \end{aligned}$$

The bubble distortion increases linearly with the film capillary number just before coalescence $Ca_f(t_c)$.

**Figure 6:**
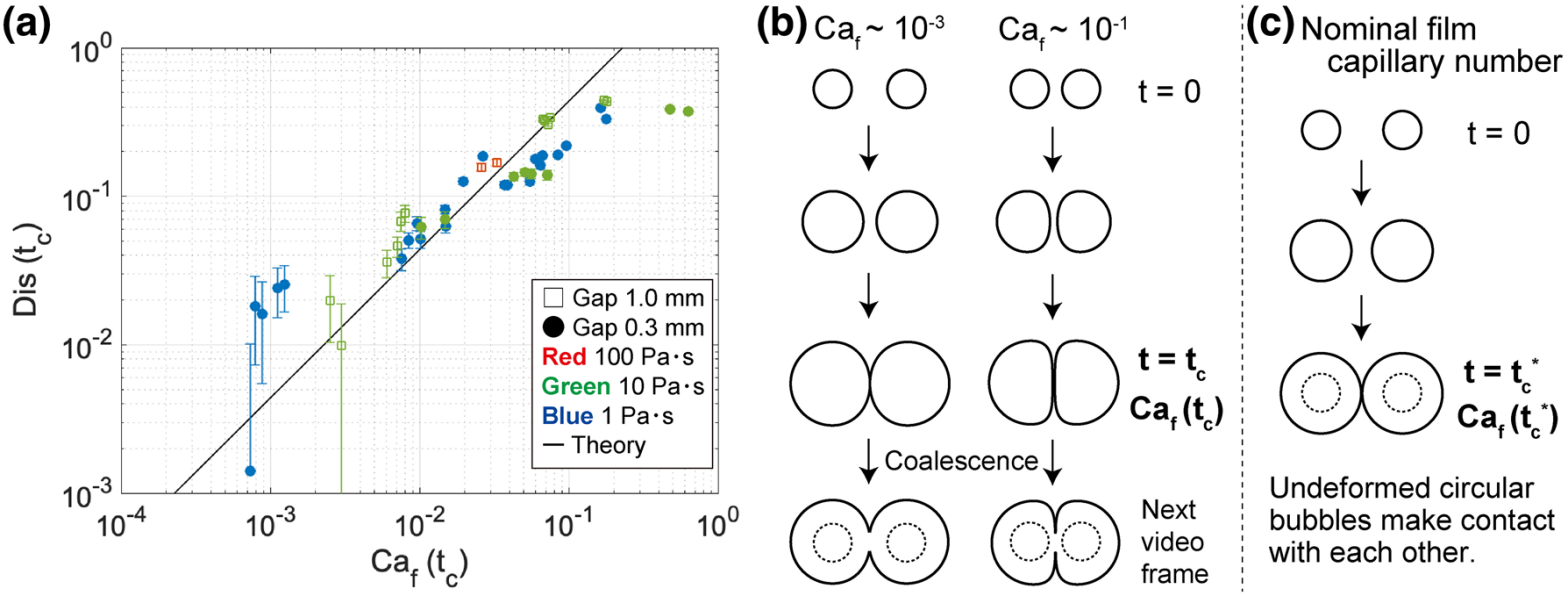
Rescaled log plot of bubble distortion just before coalescence ($Dis(t_c)$) as a function of $Ca_f(t_c)$. The solid line corresponds to the perturbation solution of Eq. (10). The error bars of bubble distortion are calculated from the spatial resolution of images. (**b**) Schematic image describing the dependence of the bubble distortion on $Ca_f$ in our experiments. (**c**) Definition of the nominal film capillary number $Ca_f(t^*_c)$.

The experimental data of $Dis(t_c)$ are replotted as a function of $Ca_f(t_c)$ in Fig. 6a. The coalescence time $t_c$ was determined by the experimental movie, and the bubble radius $R(t_c)$ and the growth rate ${\dot{R}}(t_c)$ at that time were calculated based on the image analysis. Figure 6 shows that the bubble shape before coalescence is determined solely by the film capillary number irrespective of the initial $h_{00}$, pressure reduction, and decompression rate. The linear increase of $Dis(t_c)$ with $Ca_f(t_c)$ is roughly confirmed in the range $10^{-4}<Ca_f(t_c)<10^{-1}$. We also find that the bubble distortion is independent of $Ca_f(t_c)$ for $10^{-1}<Ca_f(t_c)$. This shift of trend indicates the upper limit of the application range of our model. The perturbation solution (Eq. 10) was derived in the limit in which the viscous pressure inside a film was smaller than interfacial tension and the bubbles maintained an approximately circular shape. This limit corresponds to the small $Ca_f(t_c)$ region where a bubble does not deviate significantly from the original circular shape. In the large $Ca_f$ region, the pressure inside the film is so large that the limitation of the approximately circular bubble cannot be applied (Fig. 6b). In addition, when deriving the analytical solution we assume that the bubble centroids are fixed in space, but the experimental movie shows that the bubble centroids actually move away from each other (see Fig. S4 in the supplementary information). Moving away from another bubble reduces the bubble distortion during film drainage.

### A regime diagram of coalescence

We have dealt with only the data in which bubbles coalesced during the linear-decompression region. Hereinafter, we summarize all of the experimental data and make a regime diagram of coalescence. The coalescence condition is seemingly determined by the degree to which the bubbles distort when they come close to each other. Non-coalescence of proximal bubbles can be ascribed to the transition from a circular shape to a flat shape as they grow. In order to estimate hydrodynamic interactions of two proximal bubbles, we use a nominal film capillary number $Ca_f(t^*_c)$ defined as a film capillary number evaluated at the time when undeformed circular bubbles contact each other (Fig. 6c)^34^.

In our experiments, two bubbles have slightly different sizes and their growth rates vary with time. The nominal film capillary number can be defined as

11 $$\begin{aligned} Ca_f(t^*_c) = \frac{\eta \dot{{\bar{R}}}^*(t^*_c)}{\sigma }\frac{{\bar{R}}^*(t^*_c)^2}{D^2}, \end{aligned}$$

where $\dot{{\bar{R}}}^*$ and ${\bar{R}}^*$ are the average bubble growth rate and the average bubble radius calculated by the growth model, respectively. The timing of two undeformed circular bubbles that come into contact $t^*_c$ satisfies the following equation:

12 $$\begin{aligned} 2h_{00}(0)= & {} \int ^{t_c^*}_0 {\dot{R}}^*_1(t) dt + \int ^{t_c^*}_0 {\dot{R}}^*_2(t) dt, \end{aligned}$$

where ${\dot{R}}^*$ is the bubble growth rate given by the growth model (Eq. S9 in the supplementary information). The subscripts 1 and 2 indicate the first and second bubbles, respectively. The average bubble growth rate $\dot{{\bar{R}}}^*$ in Eq. (11) is given by $\dot{{\bar{R}}}^*=1/2 \left( {\dot{R}}^*_1 + {\dot{R}}^*_2 \right)$.

**Figure 7:**
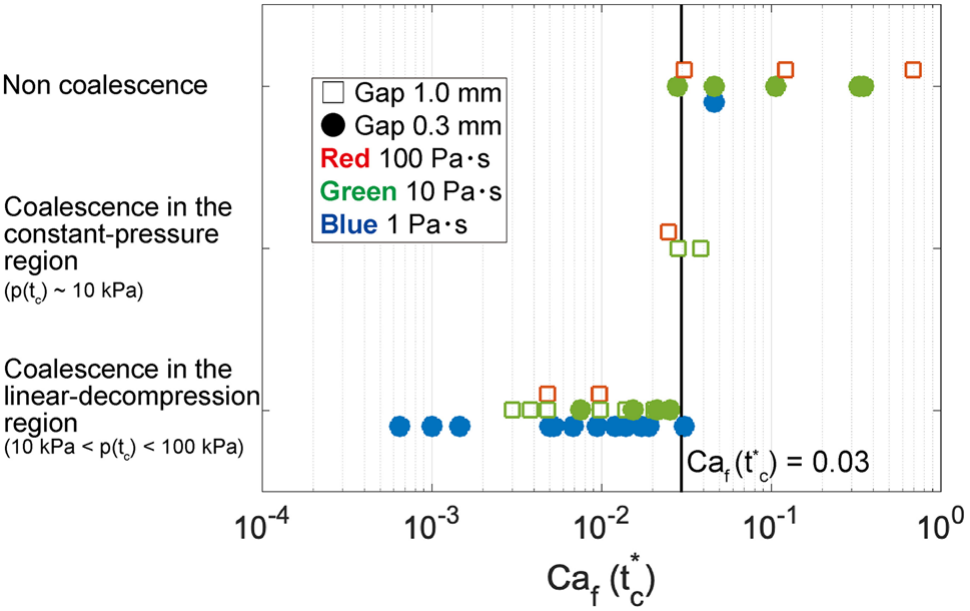
Regime diagram of bubble coalescence as a function of the nominal film capillary number $Ca_f (t^*_c)$. The black solid line indicates $Ca_f (t^*_c)=0.03$. The measured pressure at the timing of coalescence is represented by $p(t_c)$.

Figure 7 summarizes the occurrence of bubble coalescence. When the nominal film capillary number exceeds 0.03, coalescence does not occur in most of the experiments. The coalescence during the constant-pressure region occurs in the transition between coalescence and non-coalescence. There are two advantages of using the nominal film capillary number ($Ca_f(t^*_c)$) instead of the film capillary number ($Ca_f(t_c)$). First, the nominal film capillary number can be defined for both coalesced and non coalesced bubbles. Second, it does not require the measurement data, such as the temporal data of growth rate and film thickness. Thanks to these advantages, before starting the decompression, we can know whether two bubbles coalesce. We here note that the maximum pressure drop was constant in our experiments. The critical value for coalescence of $Ca_f(t^*_c)=0.03$ found in our experiments could vary slightly with the pressure drop.

## Discussion

We have considered the condition of bubble coalescence and the bubble shape just before coalescence. The film capillary number controls the dynamics of bubble film, and the nominal film capillary number predicts the occurrence of coalescence; those represent the balance between the pressure for film drainage and the interfacial stress. In volcanology, the occurrence of coalescence has been judged with the timescale of film drainage based on a parallel film model^4,35^. If the timescale of film drainage estimated by the model is shorter than the timescale of foaming from bubble nucleation to solidification, then the bubbles are assumed to coalesce. In the parallel film model, a disk film is sandwiched by two bubbles and the drainage of liquid is driven by the pressure difference between the center and margin of the film. The capillary pressure is often taken as $2 \sigma /R$ for simplicity. The parallel film model appears to be attractive in that it can derive the analytical timescale, but its applicability to practical situations is limited. This model is only available in the case that the parallel film forms between bubbles and requires the film radius and the pressure difference a priori. However, two bubbles do not always make a flat film between them, as shown in Fig. 3. Furthermore, the film radius and the curvature of film that controls the pressure difference vary significantly during bubble growth. As a previous study pointed out^32^, these deficiencies limit the usefulness of the parallel film model.

What is essential to the condition of coalescence of two growing bubbles is the deformation when they become close to each other. The bubbles maintain the circular shape and coalesce if the pressure in the film is smaller than the interfacial tension; otherwise the bubbles become distorted and do not coalesce. Since the bubble distortion results from hydrodynamic interactions between the bubble and the film (Fig. 6), the coalescence condition is determined primarily by the nominal capillary number defined as the $Ca_f(t^*_c)$, as illustrated in Fig. 7.

As an application of our experiments to a practical situation, we compare our experiments to another experiment in which bubbles are confined in a thin cell and coalesce during growth. Masotta et al. performed heating experiments of magma in a thin moissanite cell and observed the coalescence of bubbles during diffusive growth^19^. Despite the large viscosity of liquid phase ($2.5 \times 10^5 \ \mathrm{Pa \cdot s}$)^36^, the bubbles coalesced and maintained their circular shape until coalescence. Our study suggests that the bubble shape depends on the film capillary number, which takes into account not only viscosity but also bubble growth rate. The film capillary number in Masotta et al. was $Ca_f<5 \times 10^{-4}$. The low $Ca_f$ was due to the small bubble growth rate (${\dot{R}} \approx 5 \times 10^{-7} \ \mathrm{mm/s}$) and the small bubble radius ($R <1.5 \times 10^{-2} \ \mathrm{mm}$). The viscous forces in this range of $Ca_f$ are so small that bubbles can coalesce and maintain their circular shape even when the bubbles are very close (Figs. 6 and 7).

Our experiments and analysis may give a clue to the mechanism of violent volcanic eruptions of low-viscosity magma, which is a hot topic in recent volcanology^37^. Since the rapid growth of bubbles under the high decompression rate prevents coalescence, outgassing through interconnected bubbles is impeded. We also think that the suppression of bubble coalescence due to the rapid decompression may be a key factor in producing reticulites in Hawaiian eruptions. Reticulite is a basaltic pyroclast characterized by polyhedral bubbles and high vesicularities of 95–99% that are much higher than common scoria pyroclasts^38,39^. If the decompression rate of magma is high enough to prevent bubbles from coalescence, the rapid growth of bubbles generates a ’dry’ foam with polyhedral bubbles. Adiabatically expanding volcanic gas released from the bubbles cools the magma surface and enables the reticulite to fragment^40^. Since our experiments deal with the coalescence of bubbles in the quasi-two-dimensional space, it is difficult to estimate the decompression rates of reticulites whose bubbles coalesce in the three-dimensional space. In addition, a bubble in the foam is surrounded by multiple bubbles. However, the requirement of rapid decompression is supported by the fact that reticulites are found in Hawaiian high-fountain episodes^39^.

Finally, we discuss the difference between the hydrodynamic interaction in the Hele-Shaw cell and three-dimensional space, which is the real situation of foaming processes in nature and engineering. The biggest difference is the origin of viscous resistance. In our experiments, the pressure in a film arises from the viscous resistance across the cell. In other words, the pressure results from simple shear in the Poiseuille flow bound by the two plates. On the other hand, the three-dimensional bubbles are free of boundary walls. Although there have been debates about the boundary condition between gas and liquid^32^, a previous coalescence experiment using viscous liquid suggests that bubbles have fully mobile surfaces that cannot sustain the stress of simple shear across the film^5^. We expect that the film pressure in a three-dimensional foam arises from pure shear along the drainage direction, and the coalescence behavior will depend on the capillary number $Ca=\eta {\dot{R}}/\sigma$ having one characteristic length. The same capillary number was proposed in a previous study, and its utility was validated by a numerical simulation of expansion of a two-dimensional foam^34^.

Despite the above differences, our present experiments may be extended to three-dimensional coalescence in a straightforward way because the evolution of the film shape between growing bubbles may be essentially controlled by the competition between viscous force and interfacial tension. In addition, our scaling analysis and perturbation solution are useful to interpret in-situ foaming experiments that bind a bubbly liquid with a thin cell.

Thus far, we considered two bubbles growing in a Hele-Shaw cell. If multiple bubbles grow largely and the viscous forces in films are large ($1 \ll Ca_f$), a parallel film will form because the direction of bubble growth is confined due to interactions from other bubbles^34^. The multiple interactions definitely affect the evolution of the film and the coalescence behavior. We should investigate the hydrodynamic interactions in a highly vesiculated foam in the near future.

## Conclusion

We have shown the importance of hydrodynamic interactions on bubble coalescence through the decompression experiments of two bubbles in a Hele-Shaw cell. The bubble growth was mainly controlled by expansion rather than diffusional influx in the pressure range from $10 \ \mathrm{kPa}$ to atmospheric pressure. When placing two bubbles in the cell, we focused on the bubble shape just before coalescence, which may best reflect the hydrodynamic interactions between the bubbles. The distortion of the bubble increased with a film capillary number $Ca_f$ representing the competition between the pressure in a film and the interfacial tension. We developed the analytical perturbation solution of the bubble distortion in terms of the film capillary number just before coalescence and confirmed that it had good agreement with the experimental data for small $Ca_f$. We also found that the occurrence of coalescence was explained by a nominal film capillary, which is defined as the film capillary number when undeformed bubbles contact each other. The coalescence occurred only below a critical nominal film capillary. Our findings will contribute to the interpretation of the *in-situ* foaming process in a thin cell that is used widely in natural science and engineering.

## Methods

### Experimental setup

We use silicone oil (KF-96, ShinEtsu) as a Newtonian fluid. Its surface tension $\sigma$ and density $\rho$ are slightly dependent on viscosity: (1) $\sigma =21.2 \ \mathrm{mN/m}$ and $\rho =970 \ \mathrm{kg/m^3}$ for the kinematic viscosity $\nu =1000 \ \mathrm{cs}$, (2) $\sigma =21.3 \ \mathrm{mN/m}$ and $\rho =975 \ \mathrm{kg/m^3}$ for $\nu =10000 \ \mathrm{cs}$, and (3) $\sigma =21.3 \ \mathrm{mN/m}$ and $\rho =977 \ \mathrm{kg/m^3}$ for $\nu =100000 \ \mathrm{cs}$.

The Hele-Shaw cell was constructed with two glass slides ($28 \times 48 \times 1.3 \mathrm{mm}$), which are often used as microscope slides. The cell gap *D* is controlled to be 0.3 or $1.0 \ \mathrm{mm}$ by sandwiching polycarbonate spacers between the glass slides. We used a clip to hold the two glasses and the spacers together. The cell was initially filled with silicon oil. Two bubbles were then injected by a gas-tight syringe (1701N, Hamilton). When using the $0.3 \ \mathrm{mm}$ spacers, we injected a single bubble and divided the bubble into two smaller bubbles by penetrating it with a needle made of a 0.2-mm-thick acrylic plate. The volumes of the injected bubbles are 0.7 and $1.5\ \mathrm{\mu L}$ for the cases with $D=0.3$ and $1.0 \ \mathrm{mm}$, respectively. After injecting bubbles, we placed the Hele-Shaw cell in an acrylic container.

We decompressed the container from atmospheric pressure to $10 \ \mathrm{kPa}$ using a vacuum pump. The decompression rate was controlled by a vacuum regulator (EV2100V, CKD). The pressure change between a tank and the container was measured with a pressure transducer (AP-C30, Keyence) at $20 \ \mathrm{Hz}$. The tank ($3.5 \ \mathrm{L}$) was inserted between the container and the vacuum regulator in order to decrease the pressure smoothly. If there is no tank, the pressure oscillates around the set value because the regulator cannot accurately control the pressure inside a small space like the container. We set the regulator to decrease the pressure linearly, but in the cases with rapid decompression, the decompression rate gradually slowed down, as shown in Fig. 2a. We define the decompression rate *dp*/*dt* using the data for which pressure decreased linearly. When the pressure reached the final pressure ($10 \ \mathrm{kPa}$), we kept it constant for $60 \ \mathrm{s}$ and then returned the pressure to atmospheric pressure. The room temperature was set to $20^\circ \mathrm{C}$.

The sequence of bubble coalescence was recorded with a digital microscope (LM207, LINKMICRO) that captured 30 frames per second with a resolution of $1,920 \times 1,080$ pixels. The spatial resolutions for each run ranged from 7 to $14 \ \mathrm{\mu m/pixel}$. The interface between the gas and the liquid was identified by the intensity of brightness. The projected bubble area was defined as the area of the gas and the interface, and the film thickness was defined as the shortest distance between the two interfaces. The obtained bubble radius, growth rate, and film thickness include high-frequency fluctuations, which we regard as noise caused by insufficient spatial resolution. To remove the noise, we smoothed the data by taking a moving average. A supplementary data file summarizes the experimental conditions.

## Supplementary Information

Supplementary Information 1.

Supplementary Information 2.

Supplementary Information 3.

Supplementary Information 4.

Supplementary Information 5.

Supplementary Information 6.
