# Supplementary material for: Coalescence of two growing bubbles in a Hele–Shaw cell
Source: Sci Rep. 2022 Jan 24;12:1270. doi: 10.1038/s41598-022-05252-5 (PMC8786952; doi:10.1038/s41598-022-05252-5)
Supplement: Supplementary file 1 — Supplementary Information 1. [file 41598_2022_5252_MOESM1_ESM.pdf]

Coalescence of two growing bubbles in a Hele-Shaw cell

Masatoshi Ohashi[1,2], Atsushi Toramaru[2], and Atsuko Namiki[3]

[1]Earthquake Research Institute, The University of Tokyo, Yayoi, Bunkyo-ku, Tokyo, 113-0032, Japan
[2]Department of Earth and Planetary Sciences, Kyushu University, Motooka, Nishi-ku, Fukuoka, 819-0395, Japan
[3]Graduate School of Environmental Studies, Nagoya University, Furo-cho, Chikusa-ku, Nagoya,464-8601, Japan
[*]Corresponding author: m-ohashi@eri.u-tokyo.ac.jp

Experimental dataset

Supplementary dataset 1. The dataset of the experimental conditions.

This file describes the experimental conditions of all cases.

Experimental movies

Supplementary Movie 1. The movie of the decompression rate for $dp/dt = -1.5$ kPa/s.

The experimental name is 2021053104. This movie is at 8x speed.

Supplementary Movie 2. The movie of the decompression rate for $dp/dt = -0.3$ kPa/s.

The experimental name is 2021050506. This movie is at 30x speed.

Supplementary Movie 3. The movie of the decompression rate for $dp/dt = -9.0$ kPa/s.

The experimental name is 2021053108. This movie is at 4x speed.

Supplementary Movie 4. The movie of non coalescence.

The experimental name is 2021042205. This movie is at 8x speed.

1 Bubble growth

Given that the initial bubble radius R calculated from the projected area is larger than the cell thickness D, we expect that the bubble forms a cylindrical shape. In this study, we assume that the bubble growth rate is small enough to neglect the advection of dissolved gas due to the interface movement. For simplicity, the dissolved gases are assumed to be N_2, O_2, and CO_2, and their mole-fractions are assumed to be constant during decompression.

In cylindrical coordinate (r, ϕ, z), the diffusion equation of i-th gas component is approximated with the radial symmetry and no axial diffusion

$$\frac{\partial C_i}{\partial t} = \frac{\alpha_i}{r}\frac{\partial}{\partial r}\left(r\frac{\partial C_i}{\partial r}\right), \tag{S1}$$

where C_i and α_i are the molar fraction and the diffusivity of i-th gas component, respectively. Hereafter, the subscript $_i$ means the i-th gas component. The initial and boundary conditions are given by

$$C_i(R, t) = C_{s,i}(t), \tag{S2}$$

$$C_i(\infty, t) = C_i(r > R, 0) = C_{\infty,i} \tag{S3}$$

where $C_{s,i}$ and $C_{\infty,i}$ are the saturation concentration at the interface and the initial concentration, respectively. The initial concentration corresponds to the saturation concentration equilibrium with the atmospheric pressure P_{atm}. Assuming that the pressure inside the bubble is the same as the container P, we get

$$C_{s,i} = H_i X_i P(t), \tag{S4}$$

$$C_{\infty,i} = H_i X_i P_{atm}, \tag{S5}$$

where H_i is the Henry's constant and X_i is the mole fraction. Here, we assume that the temporal change of $C_{s,i}$ is slow and the bubble radius is much larger than the diffusive layer ($\alpha_i t \ll R^2$). The approximate solution of interfacial concentration gradient based on Eq. (S1) is given by [5]

$$\left.\frac{\partial C_i}{\partial r}\right|_{r=R} = \frac{(C_{\infty,i} - C_{s,i})}{\sqrt{\pi \alpha_i t}} + \frac{(C_{\infty,i} - C_{s,i})}{2R}. \tag{S6}$$

In the case of a spherical bubble in the three-dimensional space, the second term in Eq. (S6) becomes $(C_{\infty,i} - C_{s,i})/R$ [2]. The evolution of total mole number inside a bubble $\frac{dn_G}{dt}$ is given by the sum of mole number of i-th gas component $n_{G,i}$,

$$\frac{dn_G}{dt} = \sum_{i=1}^{3} \frac{dn_{G,i}}{dt} = 2\pi R D \sum_{i=1}^{3} \alpha_i (C_{\infty,i} - C_{s,i}) \left[\frac{1}{\sqrt{\pi \alpha_i t}} + \frac{1}{2R}\right]. \tag{S7}$$

By differentiating the ideal gas law with respect t, we also get $\frac{dn_G}{dt}$ as follows,

$$\frac{dn_G}{dt} = \frac{1}{R_G T}\left[2\pi R D P \frac{dR}{dt} + \pi R^2 D \frac{dP}{dt}\right], \tag{S8}$$

where R_G is the gas constant and T is the temperature. We also assume that the pressure jump across the interface is negligible. From Eqs. (S7) and (S8), the bubble growth rate is obtained,

$$\frac{dR}{dt} = -\frac{R}{2P}\frac{dP}{dt} + \sum_{i=1}^{3} \frac{\alpha_i R_G T H_i X_i \left(P_{atm} - P\right)}{P}\left[\frac{1}{\sqrt{\pi \alpha_i t}} + \frac{1}{2R}\right]. \tag{S9}$$

The first and second terms in the right-hand side represent the effect of expansion and gas diffusion, respectively. We solved Eq. (S9) numerically using a Runge-Kutta method. Parameters needed in Eq. (S9) are summarized in Table S1.

Table S1: Parameters for the bubble growth model.

Gas	Diffusivity α_i [m^2 s^{-1}][a]	Henry's constant H_i [mol Pa^{-1} m^{-3}][b]	Mole fraction X_i
N_2	3.4×10^{-9}	1.4×10^{-4}	0.78
O_2	3.4×10^{-9}	1.8×10^{-4}	0.21
CO_2	2.2×10^{-9}	1.3×10^{-3}	0.0004

[a] Diffusivity in PDMS at 35 °C [4].
[a] Henry's constant in PDMS at 20 °C [4].

2 Perturbation solution of the deformed bubble shape

We extended the method of Chan et al. [1] to the deformations of two growing bubbles confined in a Hele-Shaw cell. Figure S1 shows the schematic geometry of our model. Two cylindrical bubbles are placed in a Hele-Shaw cell in the xy-plane with cell gap D in the z direction. The undeformed bubbles have the radius R in the xy-plane that is much larger than D. The two bubbles are separated by a thin film with a thickness of $2h(x,t)$, and the pressure in the film is given by $p(x,t)$. One of the strong assumptions in our method is to neglect the motion of the bubble centroids and fix them in space. We revisit this validity in Fig. S4.

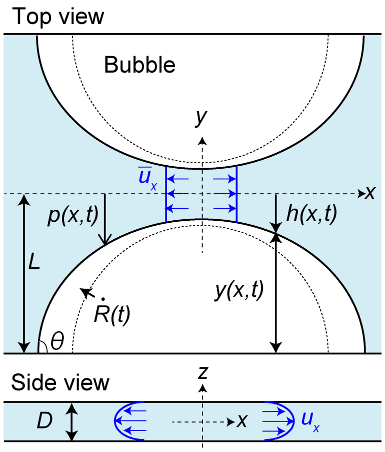

Figure S1: Schematic image of two growing bubbles in a Hele-Shaw cell with film thickness $2h$. The bubble centroids are assumed to be fixed to space. The bubbles are deformed by the pressure arising from bubble growth. The side view is the cross-section at $y = 0$.

2.1 Augmented Young-Laplace equation

The bubble height $y(x,t)$ can be obtained from the augmented Young-Laplace equation that describes the equilibrium bubble shape. In the Hele-Shaw cell, it is given by [6],

$$\sigma\left(\frac{2}{D} - \frac{\partial}{\partial x}\left[\frac{\partial y/\partial x}{\left[1 + (\partial y/\partial x)^2\right]^{1/2}}\right]\right) = \frac{2\sigma}{D} + \frac{\sigma}{R_L} - p(x,t), \tag{S10}$$

where σ is the interfacial tension and R_L is the Laplace radius determined by the conservation of bubble volume. For an undeformed bubble, R_L is equal to the bubble radius R. The Lagrange multiplier σ/R_L represents the pressure jump across the interface of a deformed bubble.

Chan et al. demonstrated the connection between the inner solution of Eq. (S10) that describes the bubble shape around the apex and the outer solution that describes the bubble deformation on the bubble scale. Through the conservation of bubble volume, they derived the inner asymptotic form of the outer solution in Eq. (S10) as

$$\begin{aligned} y_{out} &\approx R\,(1-\cos\theta) - f(t)R\left\{\frac{2 - 2\cos\theta - \theta\sin\theta}{\sin\theta - \theta\cos\theta}\right\} - \frac{x^2}{2R_L} + f(t)x \\ &= R - \frac{x^2}{2R(1-f)} - \left(2 - \frac{\pi}{2}\right) f(t)R + f(t)x, \end{aligned} \tag{S11}$$

where

$$f(t) = \frac{1}{\sigma}\int_0^R p(x,t)dx \tag{S12}$$

$$R_L = R\left\{1-\left(\frac{1-\cos\theta}{\sin\theta-\theta\cos\theta}\right)f(t)\right\} = R(1-f). \tag{S13}$$

Here, y_{out} is the outer solution of the bubble shape, f is the normalized force in a film, and θ is the contact angle (Fig. S1). For simplicity, we take $\theta = \pi/2$. The outer solution y_{out} is valid under the condition that the normalized pressure f is much smaller than the interfacial tension ($f \ll 1$). Because of this condition, it is reasonable to change the interval of integration from the original range of Chan et al. $0 \sim \infty$ to $0 \sim R$ in Eq. (S12). The first term of Eq. (S11) represents the height of the undeformed bubble, and the other terms represent the corrections to the bubble height. The outer solution should be matched with the outer asymptotic form of the inner solution. This requirement provides the boundary condition of the half-thickness h.

We now develop the inner solution of Eq. (S10) in terms of $h(x,t)$. Since we assume that the bubble centroids are fixed to the space, we obtain $L = h(x,t) + y(x,t)$, where L is the constant distance from the bubble centroid to the x axis. The augmented Young-Laplace equation can be written as a function of $h(x,t)$,

$$\sigma\frac{\partial}{\partial x}\left[\frac{\partial h/\partial x}{\left[1+(\partial h/\partial x)^2\right]^{1/2}}\right] = \frac{\sigma}{R_L} - p(x,t). \tag{S14}$$

In the present study, the pressure in a film due to bubble growth is assumed to be smaller than the interfacial tension, so that the deformation of the bubble is small on the scale of a bubble. Around the inner region $x \ll R$, it is reasonable to assume $\left|\frac{\partial h}{\partial x}\right| \ll 1$, and then we obtain a simplified Eq. (S14),

$$\sigma\frac{\partial^2 h}{\partial x^2} = \frac{\sigma}{R_L} - p(x,t). \tag{S15}$$

The assumption of small deformation suggests that the half-thickness has a perturbation form,

$$h(x,t) = h_0(x,t) + h_1(x,t) \tag{S16}$$

$$h_0(x,t) = h_{00}(t) + \frac{x^2}{2R_L}, \tag{S17}$$

where the h_0 is the zeroth-order solution of the unperturbated circular bubble, h_{00} is the half-thickness of the unperturbated circular bubble at $x = 0$, and h_1 is the first-order correction term arising from hydrodynamic interactions. Here, we approximate the circular shape to be parabolic (Eq. S17). To solve Eq. (S15) with Eq. (S16), we need the pressure distribution in the film $p(x,t)$. For Eq. (S15), we use the zeroth-order pressure p_0 arising from the growing circular bubbles.

2.2 Pressure based on a lubrication approximation

We calculate p_0 by a lubrication theory [3], assuming the flow in a film to be quasi-steady. Figure S2 shows the schematic geometry of two circular growing bubbles.

Since $h_0 \ll R$ and $D \ll R$, the flow is approximately unidirectional in the x direction. The Stokes equation are then given by

$$\frac{\partial p_0}{\partial x} = \eta\frac{\partial^2 u_x}{\partial z^2}, \tag{S18}$$

$$\frac{\partial p_0}{\partial y} = 0, \tag{S19}$$

$$\frac{\partial p_0}{\partial z} = 0, \tag{S20}$$

where u_x is the velocity in the x direction and η is the viscosity of liquid. The above equations suggest the pressure is constant across the film and the cell and depends only on the x coordinate. By integrating Eq.

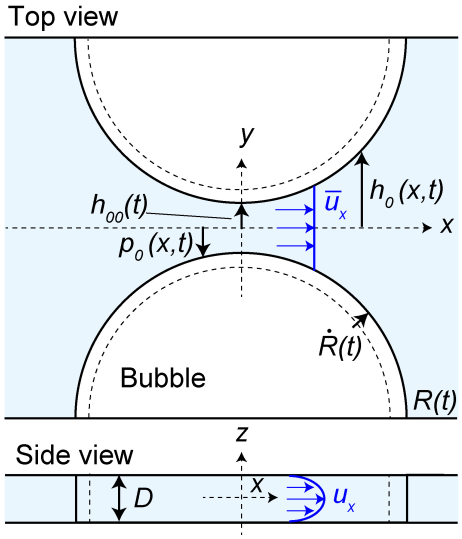

Figure S2: Schematic image of the interface of two growing bubbles confined in a Hele-Shae cell. Side view is the cross section in the xz plane along the x axis.

(S18) with the no-slip condition on the cell ($u_x|_{z=\pm D/2} = 0$), we obtain

$$u_x = \frac{\left(z^2 - D^2/4\right)}{2\eta} \frac{\partial p_0}{\partial x}. \tag{S21}$$

Since the velocity depends on z, it is useful to use the velocity averaged over the z direction $\bar{u}_x$

$$\bar{u}_x = \frac{1}{D} \int_{-D/2}^{D/2} u_x dz = -\frac{D^2}{12\eta} \frac{\partial p_0}{\partial x}. \tag{S22}$$

The continuity equation is written as

$$\frac{\partial \bar{u}_x}{\partial x} + \frac{\partial \bar{u}_y}{\partial y} = 0, \tag{S23}$$

where $\bar{u}_y$ is the velocity in the y direction averaged over the z direction. Using Eqs. (S22) and (S23) with the boundary condition $\bar{u}_y|_{y=0} = 0$, we get

$$\bar{u}_y = \frac{D^2}{12\eta} \frac{\partial^2 p_0}{\partial x^2} y. \tag{S24}$$

The boundary condition at a moving bubble surface is given by [3]

$$\bar{u}_y|_{y=h_0} = \frac{\partial h_0}{\partial t} + \bar{u}_x|_{y=h_0} \frac{\partial h_0}{\partial x}. \tag{S25}$$

For simplicity, the bubble shape is approximated as the parabolic shape

$$h_0 = h_{00} + \frac{x^2}{2R}. \tag{S26}$$

From Eqs. (S22), (S24), (S25), and (S26), we obtain

$$\frac{\partial h_0}{\partial t} = \frac{D^2}{12\eta} \left\{ \frac{\partial^2 p_0}{\partial x^2} \left(h_{00} + \frac{x^2}{2R} \right) + \frac{\partial p_0}{\partial x} \frac{x}{R} \right\}. \tag{S27}$$

Because the centroids of two bubbles are fixed, $\frac{\partial h_0}{\partial t}$ is given by

$$\frac{\partial h_0}{\partial t} = \dot{h}_{00} - \frac{x^2 \dot{R}}{2R^2} = -\dot{R}\left(1 + \frac{x^2}{2R^2}\right), \tag{S28}$$

where $\dot{R}$ is the bubble growth rate. By inserting Eq. (S28) into Eq. (S27), we obtain the differential equation of p_0,

$$\left(h_{00} + \frac{x^2}{2R}\right)\frac{\partial^2 p_0}{\partial x^2} + \left(\frac{x}{R}\right)\frac{\partial p_0}{\partial x} + \frac{12\eta\dot{R}}{D^2}\left(1 + \frac{x^2}{2R^2}\right) = 0. \tag{S29}$$

We integrate Eq. (S29) with the boundary condition that $\left.\frac{\partial p_0}{\partial x}\right|_{x=0} = 0$ and $p_0(R,t) = 0$ to obtain the solution

$$p_0(x,t) = \frac{12\eta\dot{R}}{D^2}\left\{\left(R - \frac{1}{3}h_{00}\right)\log\left(\frac{R^2 + 2Rh_{00}}{x^2 + 2Rh_{00}}\right) + \frac{1}{6R}\left(R^2 - x^2\right)\right\}. \tag{S30}$$

The above equation indicates that when the bubbles grow ($\dot{R} > 0$), the pressure inside the film increases. This increase in the pressure prevents the bubbles from coalescence.

2.3 Perturbation solution of the half-thickness

Within the perturbation scheme, the normalized hydrodynamic force f_0 based on the zeroth-order pressure p_0 is calculated from Eq. (S12)

$$f_0(t) = \frac{1}{\sigma}\int_0^R p_0(x,t)dx = \frac{4\eta\dot{R}R^2}{3\sigma D^2} + \frac{8\eta\dot{R}\left(3R - h_{00}\right)}{\sigma D^2}\left\{R - \sqrt{2Rh_{00}}\arctan\left(\sqrt{\frac{R}{2h_{00}}}\right)\right\}. \tag{S31}$$

By inserting Eqs. (S16) and (S30) into Eq. (S15), the equation of inner solution reduces to

$$\sigma\frac{\partial^2 h_1}{\partial x^2} = -\frac{2\eta\dot{R}}{\sigma D^2 R}\left(R^2 - x^2\right) - \frac{4\eta\dot{R}\left(3R - h_{00}\right)}{\sigma D^2}\log\left(\frac{R^2 + 2Rh_{00}}{x^2 + 2Rh_{00}}\right). \tag{S32}$$

We integrate Eq. (S32) with the symmetry boundary condition that $\partial h_1/\partial x = 0$ at $x = 0$,

$$\frac{\partial h_1}{\partial x} = -\frac{2\eta\dot{R}}{\sigma D^2 R}\left(R^2 x - \frac{1}{3}x^3\right) - \frac{4\eta\dot{R}\left(3R - h_{00}\right)}{\sigma D^2}\left\{x\log\left(\frac{R^2 + 2Rh_{00}}{x^2 + 2Rh_{00}}\right) + 2x - 2\sqrt{2Rh_{00}}\arctan\left(\frac{x}{\sqrt{2Rh_{00}}}\right)\right\} \tag{S33}$$

Integrating Eq. (S33) needs a boundary condition. Chan et al. found it by matching the outer asymptotic form of inner solution $h(x,t)$ with the inner asymptotic form of outer solution y_{out} (Eq. S11). Here, we evaluate the outer solution y_{out} at $x = R$ and then equate it with the inner solution h at $x = R$. The obtained solution is

$$h(x,t) = h_0(x,t) + h_1(x,t) \tag{S34}$$

where

$$h_0(x,t) = h_{00}(t) + \frac{x^2}{2R(1 - f_0)} \tag{S35}$$

$$\begin{aligned} h_1(x,t) &= \left(2 - \frac{\pi}{2}\right)f_0(t)R - \frac{\eta\dot{R}}{6\sigma D^2 R}\left(6R^2x^2 - x^4\right) \\ &- \frac{4\eta\dot{R}\left(3R - h_{00}\right)}{\sigma D^2}\left\{\frac{3}{2}x^2 + \frac{1}{2}x^2\log\left(\frac{R^2 + 2Rh_{00}}{x^2 + 2Rh_{00}}\right) - 2x\sqrt{2Rh_{00}}\arctan\left(\frac{x}{\sqrt{2Rh_{00}}}\right) + Rh_{00}\log(x^2 + 2Rh_{00})\right\} \\ &- \frac{1}{2}\frac{\eta\dot{R}R^3}{\sigma D^2} - \frac{2\eta\dot{R}R^2\left(3R - h_{00}\right)}{\sigma D^2} + \frac{4\eta\dot{R}\left(3R - h_{00}\right)}{\sigma D^2}Rh_{00}\log\left(R^2 + 2Rh_{00}\right). \end{aligned} \tag{S36}$$

The half-film thickness at the film center h_{00} is given by

$$h_{00}(t) = h_{00}(0) - \dot{R}t. \tag{S37}$$

2.4 Approximate condition for bubble distortion

The schematic geometry of bubble shape is shown in Fig. S3. The minimum bubble length at $x = 0$ is expressed as $y(0,t) = R - h_1(0,t)$. When two bubbles are about to coalesce, it is reasonable to assume that the zeroth-order film thickness based on the circular bubble is approximately zero $h_{00} \to 0$. By inserting $h_{00} = 0$ and $x = 0$ into Eq. (S36), we obtain a simplified $h_1(0,t)$ that can be valid when the two bubbles are about to coalesce. Then, $y(0,t)$ is given by

$$y(0,t) = R - h_1(0,t) \approx R - \left(\frac{265}{6} - \frac{38\pi}{3}\right)\frac{\eta \dot{R} R^3}{\sigma D^2}. \tag{S38}$$

By assuming the maximum Feret diameter to be the bubble diameter ($a = 2R$), we can rewrite the bubble distortion $Dis(t_c)$ just before coalescence as

$$Dis(t_c) = \frac{a-c}{R} \approx \frac{2R - (R + y(0,t))}{R} \approx \left(\frac{265}{6} - \frac{38\pi}{2}\right)\frac{\eta \dot{R} R^2}{\sigma D^2}. \tag{S39}$$

This result indicates that the bubble distortion increases linearly with the film capillary number ($Ca_f = \frac{\eta \dot{R} R^2}{\sigma D^2}$) until the assumption of $f \ll 1$ breaks.

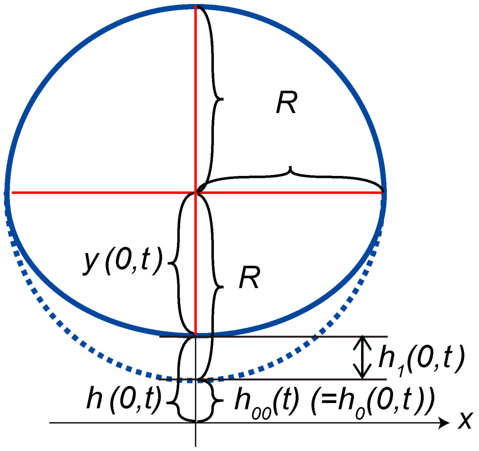

Figure S3: Schematic image of the bubble shape. Dashed line indicates the undeformed bubble shape.

3 Movement of bubbles

Figure S4a shows the outlines of two bubbles at each time. Because of the large pressure inside the film, the growth rate in the region where two bubbles face each other decreases and the bubble centroid moves away from each other. We also plot the increase rate of centroid distance just before coalescence $V(t_c)$ as a function of the average bubble growth rate just before coalescence $\bar{\dot{R}}(t_c)$ (Fig. S4b). Surprisingly, all the data points clearly indicate the linear increase of $V(t_c)$ with $\bar{\dot{R}}(t_c)$. In our experiments, the variation of bubble growth is mainly due to the decompression rate. The consideration of the movement of bubble centroids is required for modeling rapid decompression experiments of foam.

References

[1] Derek Y.C. Chan, Evert Klaseboer, and Rogerio Manica. Dynamic interactions between deformable drops in the Hele-Shaw geometry. *Soft Matter*, 6(8):1809–1815, 2010.

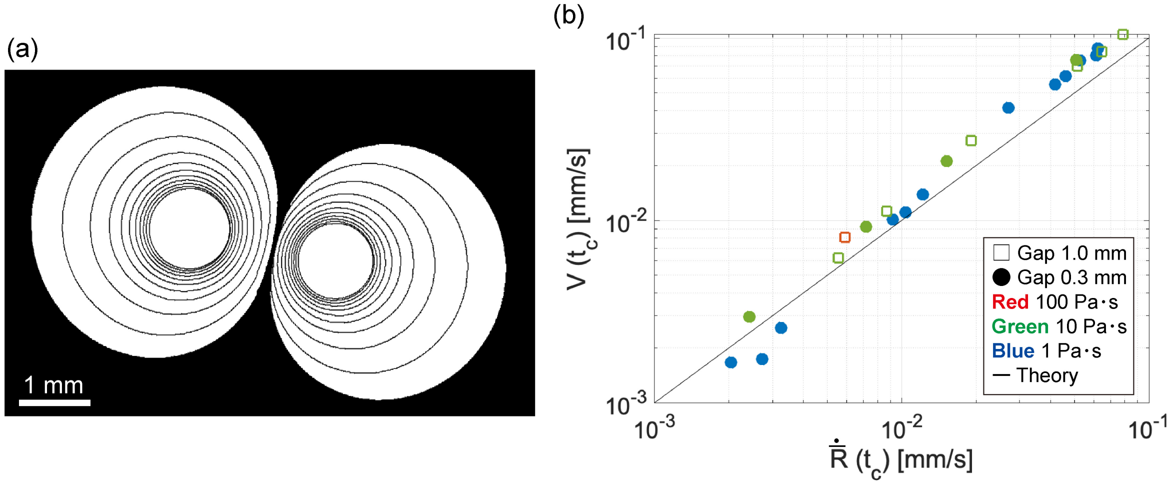

Figure S4: (a) Sequence of bubble outline with the time interval of 6.1 s (2021053104). Bubbles grow non-spherically with time. (b) Increase rate of the distance between two centroids just before coalescence $V(t_c)$ as a function of the average growth rate of two bubbles $\bar{\dot{R}}(t_c)$. All the data points roughly collapse on the theoretical line $V(t_c) = \dot{R}(t_c)$ indicated by a solid black line.

[2] P. S. Epstein and M. S. Plesset. On the Stability of Gas Bubbles in Liquid - Gas Solutions. *The Journal of Chemical Physics*, 18(11):1505, dec 1950.

[3] L. Gary. Leal. *Laminar flow and convective transport processes : scaling principles and asymptotic analysis*. Butterworth-Heinemann, 1992.

[4] T C Merkel, V I Bondar, K Nagai, B D Freeman, and I Pinnau. Gas Sorption, Diffusion, and Permeation in Poly(dimethylsiloxane) The permeability of poly(dimethylsiloxane) [PDMS] to. Technical report, 2000.

[5] Pablo Peñas-López, Benjamin Van Elburg, Miguel A. Parrales, and Javier Rodríguez-Rodríguez. Diffusion of dissolved CO2 in water propagating from a cylindrical bubble in a horizontal Hele-Shaw cell. *Physical Review Fluids*, 2(6):1–19, 2017.

[6] E. K. Yeh, John Newman, and C. J. Radke. Equilibrium configurations of liquid droplets on solid surfaces under the influence of thin-film forces. Part II. Shape calculations. *Colloids and Surfaces A: Physicochemical and Engineering Aspects*, 156(1-3):525–546, 1999.
